# Effects of collagen‐based coating with chitosan and ε‐polylysine on sensory, texture, and biochemical changes of refrigerated *Nemipterus virgatus* fillets

**DOI:** 10.1002/fsn3.3916

**Published:** 2023-12-27

**Authors:** Yongping Huang, Ying Nie, Fei Zhou, Biansheng Li, Qiulan Luo, Bin Zhang, Qinpei Zeng, Yisheng Huang

**Affiliations:** ^1^ School of Life Sciences and Food Engineering Hanshan Normal University Chaozhou China; ^2^ College of Food Science and Engineering South China University of Technology Guangzhou China; ^3^ Guangdong Wuqiong Food Group Co., LTD Chaozhou China

**Keywords:** biological preservatives, chitosan, collagen, *Nemipterus virgatus*, ε‐Polylysine

## Abstract

In order to evaluate the effects of chitosan, ε‐polylysine, and collagen on the preservation properties of refrigerated *Nemipterus virgatus*, samples were tested with different treatments for 10 days, namely chitosan, ε‐polylysine and collagen (CH + ε‐PL + CA), chitosan and ε‐polylysine (CH + ε‐PL), chitosan and collagen (CH + CA), ε‐polylysine and collagen (ε‐PL + CA), and the uncoated sample (CK). The results demonstrated that the bio‐coating exhibited better preservation effects. The CH + ε‐PL + CA, CH + ε‐PL, CH + CA, ε‐PL + CA treatments could significantly inhibit bacterial growth and retard the increase of total volatile base nitrogen (TVB‐N), 2‐thiobarbituric acid (TBA), K‐value, and total viable counts (TVC) in *N. virgatus* fillets. The pH of all samples decreased and reached its lowest value on day 6, then increased significantly at the end of the experiment (*p* < .05). Water‐holding capacity (WHC) of all the groups decreased continuously throughout storage, and CK reached 66.03% on day 6, which is significantly lower than CH + ε‐PL + CA, CH + ε‐PL, CH + CA, and ε‐PL + CA (*p* < .05). On the contrary, the sensory scores of CH + ε‐PL + CA, CH + ε‐PL, CH + CA, and ε‐PL + CA were significantly higher than the control, and the score of CH + ε‐PL + CA (*p* < .05) was the best among all the groups. In terms of texture, CH + PL + CA also showed less cell shrinkage and tighter muscle fiber arrangement compared to other treatments. To sum up, the CH + PL + CA bio‐coating proved to be a promising method for maintaining the storage quality of *N. virgatus* under refrigerated storage conditions.

## Graphical abstract

We investigated the effects of chitosan, ε‐polylysine, and collagen coatings on the preservation quality of refrigerated *Nemipterus virgatus* for 10 days. CH, ε‐PL, and CA showed a good synergistic effect with enhanced antimicrobial activity and proteolytic inhibition. CH + PL + CA performed the best in maintaining the quality of *N. virgatus* fillets.

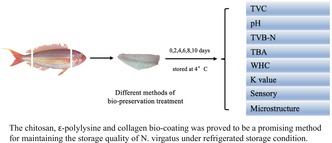

## 1 INTRODUCTION

Golden threadfin (*Nemipterus virgatus*), belonging to the genus *Nemipterus* in the family *Nemipteridae*, is a warm‐temperate zone near‐bottom fish with delicious meat and rich nutrition and is one of the most important economic fish species in Southeast Asian countries, such as China, Vietnam, Malaya, and so on (Li, Sun, et al., 2020; Li, Wang, et al., 2020). Furthermore, the preservation, transportation, and consumption of *N. virgatus* are limited by its high protein content (up to 19.7% for the whole fish) (Li, Sun, et al., 2020) and by both microbial and chemical actions, which are the main elements that cause the deterioration in the quality of fish flesh (Lemos et al., 2023; Nawaz et al., 2020; Tsoukalas et al., 2023; Yu et al., 2021). Particularly, fish spoilage can lead to many unsatisfactory quality changes, such as loss of water, softening of texture, and undesirable changes in color or other sensory attributes (Lang et al., 2023). Thus, most fish end up in low‐value products such as aquaculture, compost, and animal feeds. In order to maintain the quality and enhance the economic value of *N. virgatus* and to meet the demand of customers for fresh and safe products, it makes sense to develop an eco‐friendly and economical preservation strategy to extend the shelf life of fish.

Edible bio‐coatings made of biodegradable and edible biopolymers (e.g., polysaccharides, proteins, and lipids) have been widely used to improve the quality and shelf life of various food species during storage due to their efficiency and safety (Liu, Shibata, et al., 2020; Mohamed et al., 2020). The advantage of bio‐coating is that it delays the release of the substances added to the film, such as proteins, chitosan, bacteriocins, essential oils, and plant extracts (Chumsri et al., 2022). Chitosan (CH) is an alkaline amino‐polysaccharide obtained by deacetylation of chitin and has been widely used in food preservation due to its biodegradability, biocompatibility, and non‐toxicity (Abdollahzadeh et al., 2023; Jiang et al., 2018; Wang et al., 2023). Studies have shown that CH delays quality deterioration and prolongs shelf life to a certain extent by controlling the growth of microorganisms in aquatic products (Bonilla et al., 2019). ε‐Polylysine hydrochloride (PL) is a microbial‐derived water‐soluble peptide with antibacterial activity, consisting of 25–30 residues of L‐lysine linked by a peptide bond between the ε‐amino group and the α‐carboxylic group (Jia, Li, Fang, & Chen, 2019; Wang et al., 2021). Furthermore, the PL exhibits high efficiency and broad‐spectrum bacteriostasis, good solubilities and stability, and safety (Hiraki et al., 2003; Yang et al., 2020). Collagen, a typical macromolecule naturally present in the skins and leather of mammals (bovine and porcine), poultry, and fish, is widely used as an edible coating material due to its biodegradable, non‐toxic, and non‐antigenic properties (Liu et al., 2015; Liu, Shibata, et al., 2020). Furthermore, it has been exploited as a coating material due to its high mechanical properties, oil resistance, oxygen barrier, biocompatibility, and potential use as a carrier of different compounds and additives (Khodaei et al., 2021; Liu, Shibata, et al., 2020).

Previous studies on the application of CH, CA, or ε‐PL alone in food preservation have been widely reported. However, to the best of our knowledge, no published data currently exists on the use of a CH + ε‐PL + CA coating enriched with high mechanical, natural antioxidant, and antimicrobial properties to prolong the shelf life of fresh fish. Therefore, our work aimed to evaluate the effect of CH + ε‐PL + CA, CH + CA, CH + ε‐PL, and ε‐PL + CA bio‐coatings on texture, microbiological, chemical, and flavor quality of *N. virgatus* fillets during refrigerated storage. The study provides essential data for the interactions between CH, CA, and ε‐PL, as well as a feasible way to make eco‐friendly antimicrobial edible coatings.

## 2 MATERIALS AND METHODS

### 2.1 Samples and chemicals

Fresh *Nemipterus virgatus* were purchased from a local aquatic market in Chaozhou, China, and transferred to the laboratory in water within 30 min. The mean weight and length of *N. virgatus* were roughly 510 g and 40 cm, respectively. *N. virgatus* were beheaded, scaled, gutted, and filleted, and then washed immediately with cold, sterile water. The fillets were then drained at 4°C for 3 min and prepared for biological preservative coating.

The fillets (112 ± 14 g, about 11 × 5 × 1.5 cm) were randomly divided into six batches (6 fish per batch) for (a) CH + ε‐PL + CA (coated with CH + ε‐PL + CA coating solution); (b) CH + ε‐PL (coated with CH + ε‐PL coating solution); (c) CH + CA (coated with CH + CA coating solution); (d) ε‐PL + CA (coated with ε‐PL + CA coating solution); and (e) CK (uncoated).

**FIGURE 1:**
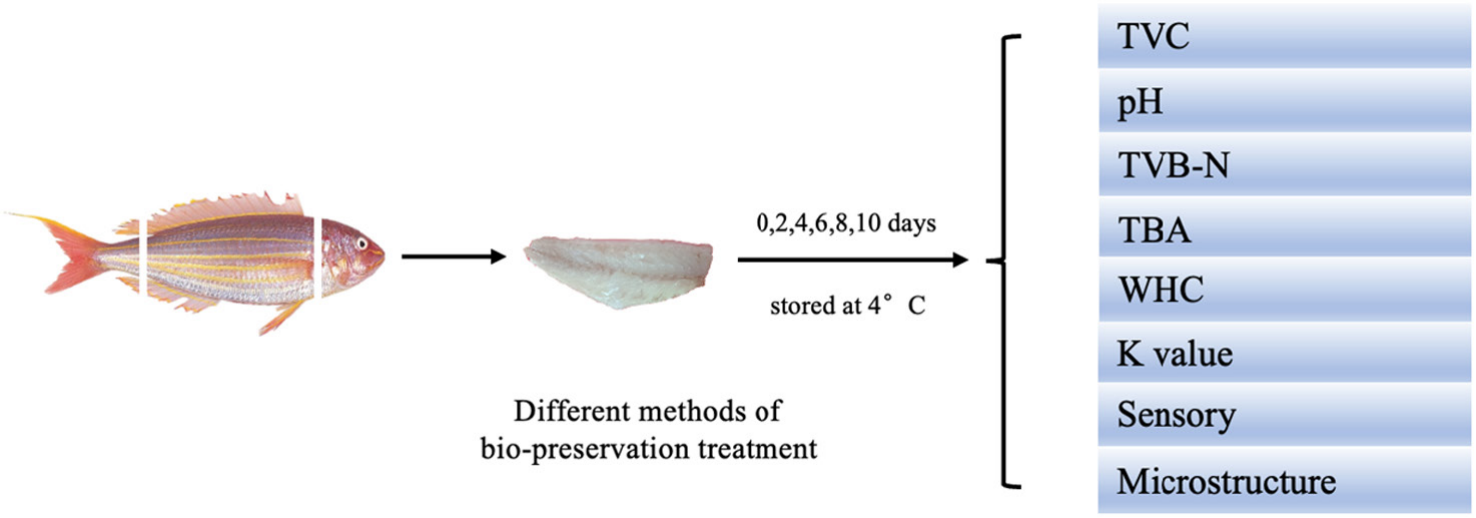
Experimental design diagram.

Each batch of *N. virgatus* was immersed in the corresponding freshly prepared coating solutions for 15 min at 4°C with a solution ratio of 1:2 (w/v). Then the *N. virgatus* samples were placed in a sterile biochemical incubator with medium airflow for 50 min at 4°C to form the bioactive coating. The *N. virgatus* samples were individually packaged in sterile freezer bags afterward and stored at 4°C until analysis. The samples were randomly taken out for analysis on the 0, 2nd, 4th, 6th, 8th, and 10th days (Figure 1).

### 2.2 Sensory evaluation

The quality of *N. virgatus* was graded according to the method of Cardenas (Bonilla et al., 2007) with some modification, and sensory scores were graded by a well‐trained panel of 10 evaluators. Each evaluator accessed the characteristics from 1 (the lowest quality) to 10 (the highest value) with regard to color, odor, texture, and overall acceptability of the test samples. The fish was considered acceptable until the sensory score was under 5.0.

### 2.3 Total viable counts

Whole, fresh gold thread samples were cut into small pieces. A 25 g sample was transferred aseptically to a sterile stomacher bag and diluted 10 times in a physiological saline solution (0.85% NaCl). The mixture was homogenized for 1 min using a stomacher to obtain the first dilution, from which successive decimal dilutions were prepared. The total viable counts (TVCs) were incubated at 30°C for 48 h, counted, and expressed as log10 colony‐forming units (CFU)/g.

### 2.4 pH and TVB‐N

The pH value was detected using a digital pH meter (LC‐Ph‐3S, Shanghai, China). TVB‐N was measured according to the Chinese National Standard (GB 5009.228–2016). Briefly, 10 g of fish sample was mixed with 75 mL of distilled water for 30 min in a conical flask, followed by homogenization and filtering. 5 mL of the filtrate and 5 mL of an MgO suspension were transferred into a reaction chamber. Then, a boric acid solution (10 mL) and a mixture of methyl red and methylene blue (v:v = 2:1) were injected, followed by distillation. Subsequently, the above solution was titrated with a 0.1 M HCl solution. The TVB‐N value, which was expressed in mg nitrogen (mg/100 g) 100 g^−1^ of fish sample, was calculated by the titration volume of the HCl solution.

### 2.5 Water‐holding capacity

Appropriately 3 g (W_1_) of minced fillet samples were prepared for water‐holding capacity (WHC) tests. The fillets were completely wrapped in a double layer of filter paper, centrifuged at 2264 *g* for 10 min, and then weighed (W_0_) after centrifugation. The WHC was calculated according to the following formula: WHC = W_0_/W_1_ × 100%.

### 2.6 *K*‐value

*K*‐value was determined according to methods proposed by Karim et al. (2019). ATP‐related compounds were analyzed using HPLC (1260 infinity; Agilent). *K*‐value was calculated as follows:

$$K\;\text{value}\;\left(\%\right)=\frac{\mathrm{HxR}+\mathrm{Hx}}{\mathrm{ATP}+\mathrm{ADP}+\mathrm{AMP}+\mathrm{IMP}+\mathrm{HxR}+\mathrm{Hx}}\times 100$$

### 2.7 Microstructure analysis

Microstructure analysis sample preparations for microscopic analysis were conducted according to HE staining routine protocols (Bonilla et al., 2007), with some modification for *N. virgatus* fillets, to observe extra‐cellular space and fiber shrinking in tissue. After undergoing deparaffinization and rehydration, 5 𝜇m longitudinal tissue sections were subjected to a series of staining steps. Initially, the sections were immersed in a hematoxylin solution for 5 min, followed by 5 dips in 1% acid ethanol (1% HCl in 70% ethanol), and subsequently rinsed with distilled water. Next, the sections were stained with an eosin solution for 3 min, followed by a dehydration process using graded alcohol and clearing in xylene. Finally, the prepared slides were meticulously examined and captured using an Olympus BX53 fluorescence microscope (Tokyo, Japan).

### 2.8 Statistical analysis

All data were subjected to one‐way analysis of variance (ANOVA) and expressed as the mean ± standard deviation (n = 3). When there were significant differences (*p* < .05), the group means were further compared with Duncan's multiple‐range tests. Statistical analyses were performed using SPSS 20.0 (SPSS, Chicago, IL, USA).

## 3 RESULTS AND DISCUSSION

### 3.1 Total viable counts

**FIGURE 2:**
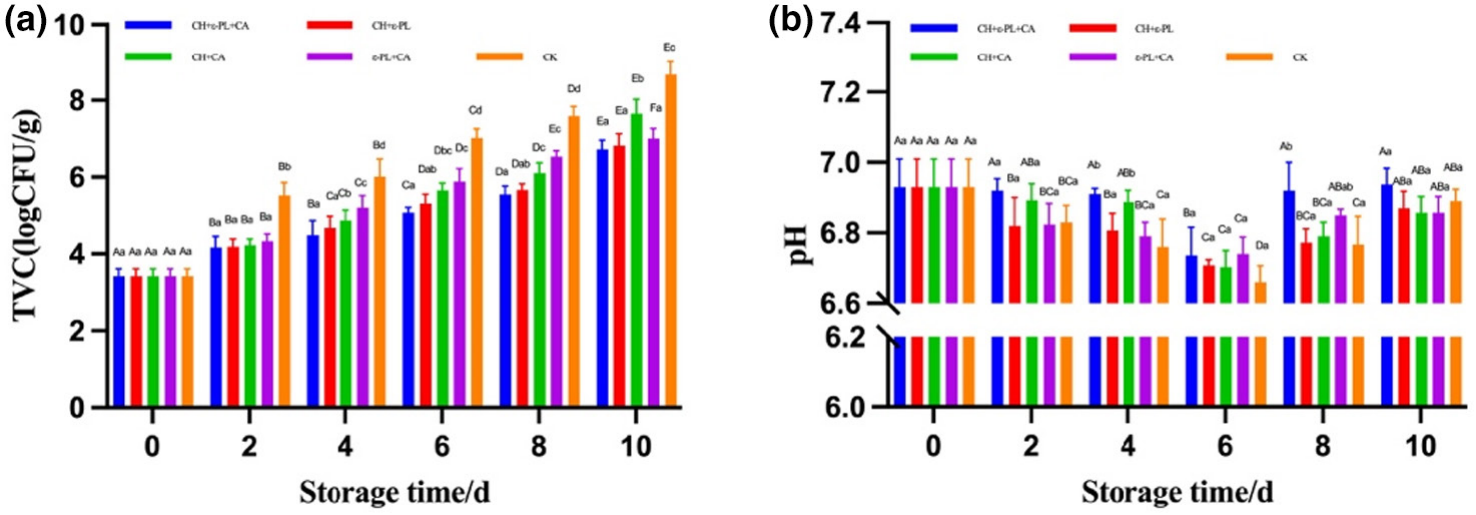
Changes in total viable counts (a) and pH (b) of *Nemipterus virgatus* during cold storage. Bars represent the standard deviation (*n* = 3). (**CH + ε‐PL + CA**: immersed in chitosan, ε‐polylysine, and collagen bioactive coating solution; **CH + ε‐PL**: immersed in chitosan and ε‐polylysine bioactive coating solution; **CH + CA**: immersed in chitosan and collagen bioactive coating solution; **ε‐PL + CA**: immersed in ε‐polylysine and collagen bioactive coating solution; **CK**: control check). Different capital letters indicate significant differences between groups, and different lowercase letters indicate significant differences within groups (*p* < .05).

As shown in Figure 2a, the TVC of all treatments increased across the course of refrigerated storage. The initial TVC ranged from 3.14 to 3.32 log CFU/g, indicating the good quality of *N. virgatus* in consideration that the maximum acceptable count for fresh fish is 5 log CFU/g (Zheng et al., 2020). In the present study, the TVC increased significantly with prolonged storage time for all samples (*p* < .05), with CH + ε‐PL + CA performing the lowest TVC proliferation. At the end of the storage, the TVC levels of the CH + ε‐PL + CA, CH + ε‐PL, CH + CA, and ε‐PL + CA groups were significantly lower than the uncoated sample. The results were considered to be related to the antibacterial properties of CH and ε‐PL as well as the antioxidant capacity of CA coating, which may play a pivotal role in rendering the bacterial population dormant and inhibiting microbial activity during the sample preparatory stage (Chumsri et al., 2022). The acceptable TVC limit for fish is recommended at 7.0 log CFU/g (ICMSF, 2002). The TVC of the uncoated sample exceeded the upper limit on Day 6, the CH + CA and ε‐PL + CA groups exceeded the limit until Day 10, while the CH + ε‐PL and CH + ε‐PL + CA groups were under the limit throughout storage. The results were presumably due to the antioxidant and antibacterial effects of CH, ε‐PL, and CA, and the coating was thus proved to extend the shelf life of *N. virgatus*, and TVC could be used as one of the evaluation criteria.

### 3.2 pH

Figure 2b displays how the pH of the fillets changed throughout storage. The pH of the uncoated fillet sample, together with those coated with CH + ε‐PL + CA, CH + ε‐PL, CH + CA, and ε‐PL + CA, decreased significantly during the first 6 days and increased significantly at the end of storage (*p* < .05). There was no significant difference in pH value on the 6th day, though the coated sample tended to retain higher pH values (*p* > .05). The generation and accumulation of acidic compounds such as lactic acid and phosphoric acid during storage might have caused the decrease in pH (Li et al., 2021; Yu et al., 2017). On the other hand, the accumulation of volatile substances such as ammonia, trimethylamine, and alkaline matter from the activity of microorganisms and endogenous enzymes will cause an increase in pH (Liu, Lan, et al., 2020). The results indicated that biological coating could effectively delay the development of basic and acidic molecules as well as maintain the quality of *N. virgatus*, acting as a buffer during storage (Chumsri et al., 2022), which might be due to the barrier property of the CA coating and the antibacterial activity of CH and ε‐PL (Li et al., 2021).

### 3.3 TVB‐N

**FIGURE 3:**
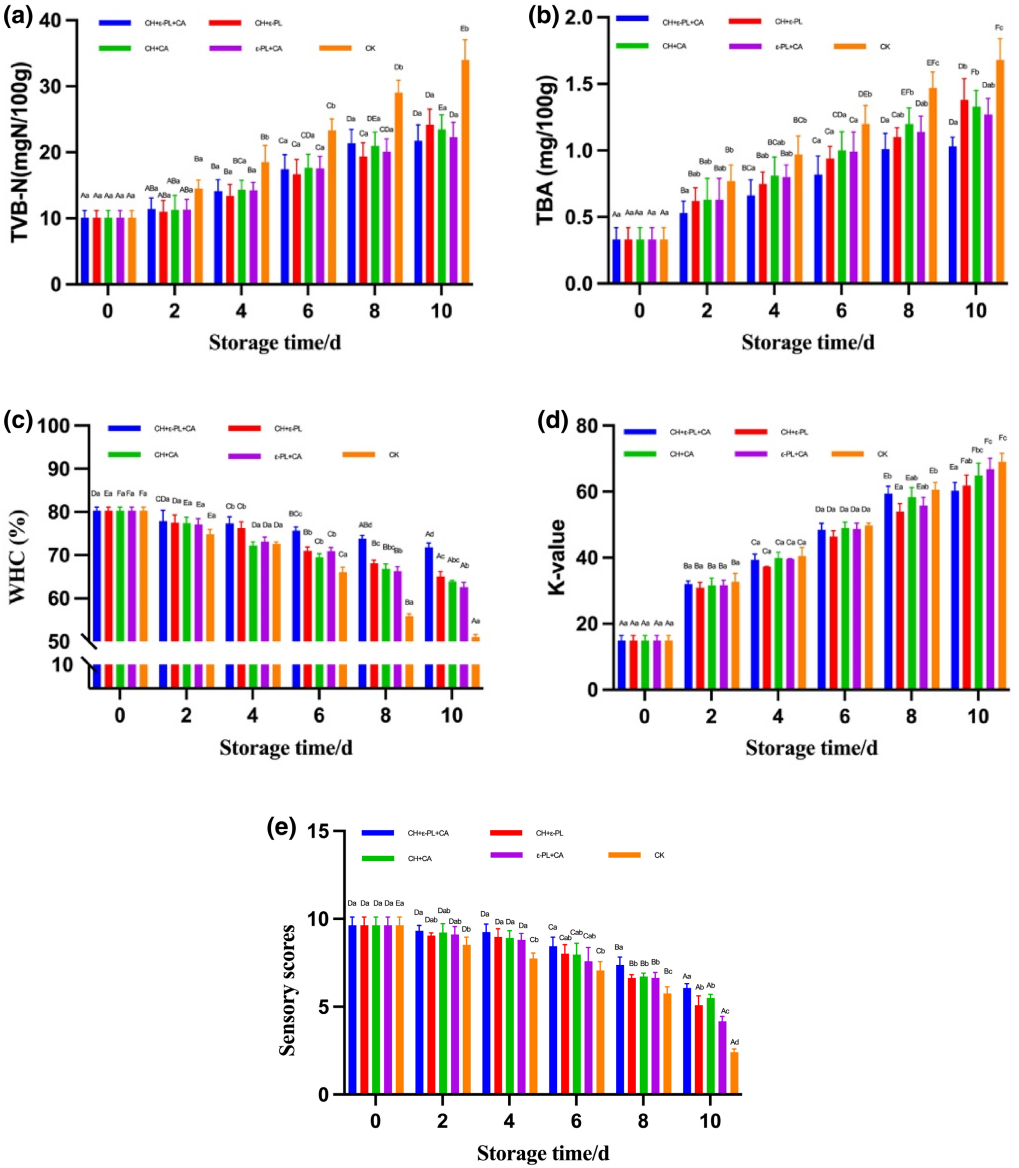
Changes in TVB‐N (a), TBA (b), WHC (c), *K*‐value (d), and sensory scores (e) of *Nemipterus virgatus* during cold storage. Bars represent the standard deviation (*n* = 3). (**CH + ε‐PL + CA**: immersed in chitosan, ε‐polylysine, and collagen bioactive coating solution; **CH + ε‐PL**: immersed in chitosan and ε‐polylysine bioactive coating solution; **CH + CA**: immersed in chitosan and collagen bioactive coating solution; **ε‐PL + CA**: immersed in ε‐polylysine and collagen bioactive coating solution; **CK**: control check). Different capital letters indicate significant differences between groups, and different lowercase letters indicate significant differences within groups (*p* < .05).

TVB‐N is an important parameter relating to the degree of protein decomposition by enzymes and microorganisms, which was recommended as a significant indicator of the freshness of the fish (Volpe et al., 2015). Figure 3a shows the increase in TVB‐N value throughout storage; the uncoated sample was significantly higher than others from day 4 to day 10 (*p* < .05). Protein decomposition means the accumulation of basic nitrogen‐containing substances, such as ammonia, dimethylamine, TMA, and some other volatile basic nitrogen compounds, among which TMA is produced by microbial enzymes that degrade trimethylamine oxide (TMAO) (Li et al., 2021; Zhuang et al., 2020). Combined with the sensory scores, a flavor of putrid fish appeared on day 9, which is probably due to the approach of the TVB‐N acceptable limit (Jia, Li, Zhuang, et al., 2019). Furthermore, the amounts of produced TVB‐N in CH + ε‐PL + CA were significantly less than those of other groups, indicating a slower degradation rate of protein by spoilage bacteria. The TVB‐N results could be attributed to the synergistic effect of CH, ε‐PL, and CA, and their effects on antibacterial activity and, furthermore, on the inhibition of protein decomposition, which is supported by the TVC result.

### 3.4 TBA

The TBA index is usually related to the degree of lipid oxidation in tissues, which is an important indicator reflecting the freshness of fish (Kaewprachu et al., 2017; Wu et al., 2016). In Figure 3b, the TBA values of all samples exhibited varied rates of increase throughout storage, which is consistent with the findings of mackerel *Scomber scombrus* (Trigo et al., 2022) and Asian sea bass *Lates calcarifer* (Chaijan et al., 2020). As shown in Figure 2b, the TBA levels in CH + ε‐PL + CA, CH + ε‐PL, CH + CA, and ε‐PL + CA increased significantly throughout storage, whereas the uncoated sample rose with no significant difference (*p* < .05). The initial TBA value in the uncoated sample was 0.33 mg MDA/kg, peaking at 1.68 mg MDA/kg finally, compared to 1.03, 1.38, 1.33, and 1.27 mg MDA/kg in CH + ε‐PL + CA, CH + ε‐PL, CH + CA, and ε‐PL + CA at the end of storage, respectively. It was considered that CH and CA act synergistically as antioxidants to inhibit lipid oxidation in *N. virgatus*, and CA had the best effect on slowing the oxidative rate by reducing oxygen diffusion (Chaijan et al., 2020).

### 3.5 Water‐holding capacity

Figure 3c displays the changes in water loss in the fish fillets as measured by WHC. All samples tended to decrease throughout storage (*p* < .05). The initial WHC of fish fillets was 80.23%, and it decreased to 51.07%, 71.82%, 65.07%, 63.89%, and 62.60% for the control, CH + ε‐PL + CA, CH + ε‐PL, CH + CA, and ε‐PL + CA at the end of the storage, respectively. The results were considered to be connected to the structural changes in muscle proteins due to hypothermia‐induced denaturation, proteolysis, protein oxidation, and interactions among proteins and oxidized lipid, aldehyde, amine, and oxide compounds, which led to a decrease in WHC (Chaijan et al., 2020). The relatively high WHC in the fillets represents the high quality of proteins and was believed to be linked with the anti‐denaturation effects of proteins in the presence of CH, ε‐PL, and CA substances. CH + ε‐PL + CA were significantly higher in WHC than others from day 6 to day 10. The higher level of WHC in the CH + ε‐PL + CA group further confirms the synergistic effect of CH, ε‐PL, and CA coatings on water retention and protein protection effects during refrigeration, which was also confirmed by the TBA and TVB‐N indices.

### 3.6 *K*‐value

*K*‐value is deemed an important indicator for fish freshness evaluation, which is obtained from the ATP degradation pathway ATP → ADP → AMP → IMP→HxR → Hx (Febrianto & Zhu, 2023; Prabhakar et al., 2020). During the progress of degradation, IMP imparts an umami (pleasant salty taste) flavor that contributes to the improvement of the quality of fresh fish, while its transformation in Hx and HxR produces unpleasant bitterness and off‐flavors that negatively affect fish flavor (Yu et al., 2021). As shown in Figure 3d, *K*‐values increased at varied rates in all *N. virgatus* samples through storage (*p* < .05). An increase in *K*‐value represents the deterioration of fish meat. *K*‐value is defined as the ratio of the sum of HxR and Hx to the sum of ATP‐degrading compounds (Chang et al., 2020). Furthermore, a *K*‐value between 20% and 40% indicates moderate freshness of the product, while 60% or more represents initial deterioration (Cai et al., 2014). As shown in Figure 3e, the *N. virgatus* fillets treated with CH + ε‐PL + CA, CH + ε‐PL, CH + CA, and ε‐PL + CA coatings significantly delayed the increase in *K*‐values compared with the uncoated sample, which is similar to the trend of TBA and TVB‐N. In the beginning, the average *K*‐value was 14.32%, indicating the good quality of the fish fillet. On day 4, the uncoated sample exceeded 40%, while those of CH + ε‐PL + CA, CH + ε‐PL, CH + CA, and ε‐PL + CA remained at 39.35%, 37.31%, 39.91%, and 39.66%, respectively. The *K*‐value of CH + ε‐PL + CA at day 10 was only 60.24%, which was significantly lower than those of the CH + CA, ε‐PL + CA, and CK groups (*p* < .05). In conclusion, CH and ε‐PL could effectively inhibit the degradation of ATP in *N. virgatus* in consideration of their antibacterial ability, and the synergistic effect among antioxidants and collagen was believed to further strengthen the antioxidant effects.

### 3.7 Sensory analysis

The decline in sensory score is usually related to the proliferation of microorganisms, the enzymolysis of protease, and lipid oxidation in tissue. As shown in Figure 3e, the sensory scores of all groups continuously decreased with prolonged storage time (*p* < .05). Similar trends were also reported in red porgy (*Pagrus major*) (Liu, Shibata, et al., 2020) and sea bass (*Lateolabrax japonicus*) (Liu, Lan, et al., 2020). All the sensory scores showed no significant difference (*p* > .05) on the first 6 days, except for the control sample. The score of uncoated samples dropped sharply compared with CH + ε‐PL + CA at the 2nd and 6th days (*p* < .05). On day 6, the score of uncoated samples decreased to 7.07, which indicated the beginning of rot in the fillets. The sensory score of CH + ε‐PL + CA was significantly higher than those of others from day 8 to day 10, which might be due to the fact that CH + ε‐PL + CA coating has more antibacterial and antioxidant capacity than CH, CH + CA, and ε‐PL + CA mixture. In conclusion, the sensory evaluation reflected a better microbial inhibition and quality maintenance capacity of CH + ε‐PL + CA.

### 3.8 Microstructure analysis

**FIGURE 4:**
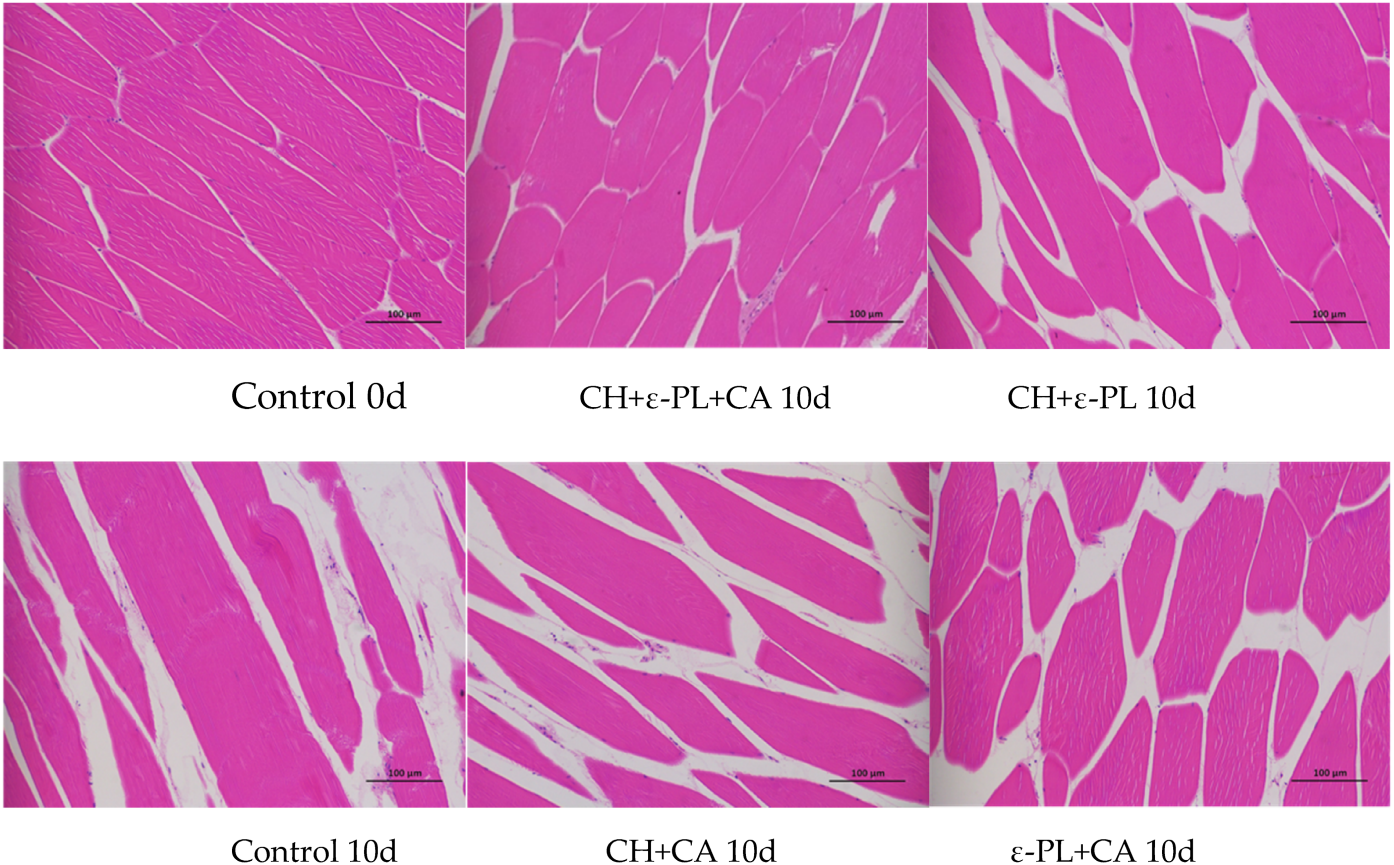
Light microscopy observations (×200) of *N. virgatus* muscle samples.

As shown in Figure 4, the structure of fresh *N. virgatus* dorsal muscle was relatively complete, the texture was clear, and the muscle fibers were closely and evenly arranged. With regard to the bio‐preservation effects, CH + ε‐PL + CA showed better results than the control, CH + ε‐PL, CH + CA, and ε‐PL + CA treatments. In the current study, the cell gap of *N. virgatus* gradually increased throughout storage, while CH + ε‐PL + CA maintained the basic tissue morphology on day 10, with relatively less cell shrinkage, tighter muscle fiber arrangement, and a clearer texture. Similar preservation effects were observed among CH + ε‐PL, CH + CA, ε‐PL + CA treatments; however, more cell shrinkage and gaps between muscle fibers were observed than those of the CH + ε‐PL + CA group. Especially in the uncoated sample, the degradation of connective tissue in the cytoskeleton results in the dispersion of muscle fiber and even breakage in some areas. In summary, the results underline that CH + ε‐PL + CA treatment has the best fresh‐keeping effect on *N. virgatus*, which is more conducive to the maintenance of muscle fiber tissue integrity.

## 4 CONCLUSION

The results of the current study concluded that CH, ε‐PL, and CA act as bio‐coatings that improve the freshness of *N. virgatus* fillets during refrigeration, ideally. The combination of CH + ε‐PL + CA was more effective than CH + ε‐PL, CH + CA, and ε‐PL + CA in terms of texture, antimicrobial, and freshness preservation effects (e.g., TVC, pH, TVB‐N, TBA, WHC, K‐value, microstructure, and sensory scores). The sensory score of CH + ε‐PL + CA was significantly higher than other groups from day 8 to day 10, and CH + ε‐PL + CA and CH + ε‐PL were under the TVC limit throughout storage compared to the control that exceeded the upper limit on day 6. CH + ε‐PL + CA, CH + ε‐PL, CH + CA, and ε‐PL + CA were significantly lower in both TVB‐N and TBA than the control, with CH + ε‐PL + CA performing the best results. The WHC of CH + ε‐PL + CA was significantly higher, while the K‐value of CH + ε‐PL + CA, was significantly lower than CH + CA, ε‐PL + CA and control at the termination of preservation. Furthermore, CH + ε‐PL + CA maintains the basic tissue morphology on the 10th day of preservation. In conclusion, the results demonstrated that CH + ε‐PL + CA could serve as an effective bio‐preservation formula for sustaining the quality and prolonging the shelf life of fish products.

## AUTHOR CONTRIBUTIONS

**Yongping Huang:** Conceptualization (equal); data curation (equal); writing – original draft (equal). **Ying Nie:** Data curation (equal). **Fei Zhou:** Resources (equal); software (equal). **BianSheng Li:** Project administration (equal). **Qiulan Luo:** Formal analysis (equal). **Bin Zhang:** Validation (equal). **Qinpei Zeng:** Resources (equal). **Yisheng Huang:** Funding acquisition (supporting); writing – review and editing (equal).

## CONFLICT OF INTEREST STATEMENT

The authors declare no conflict of interest.

## ETHICS STATEMENT

Ethical Review: This study does not involve any human or animal testing. Informed Consent: Written informed consent was obtained from all study participants.

## Data Availability

Research data are not shared.
